# Supplementary material for: SARS-CoV-2 RT-qPCR testing of pooled saliva samples: A case study of 824 asymptomatic individuals and a questionnaire survey in Japan
Source: PLoS One. 2022 May 12;17(5):e0263700. doi: 10.1371/journal.pone.0263700 (PMC9098043; doi:10.1371/journal.pone.0263700)
Supplement: S1 File — (DOCX) [file pone.0263700.s005.docx]

**Information on the PCR screening test program**

**< Eligibility for screening>**

Those who fit into both of the following items will be eligible for the screening.

1. Those who have no symptoms suggesting infectious diseases, such as fever above 37.5° C or coughing.
2. Those who have had no close contact with people with confirmed SASR-CoV-2 infection in the past two weeks.

- Note: If you are “currently having flu-like symptoms,” “have been in close contact with people who have tested positive for SARS-CoV-2 in the last two weeks,” or “have returned to Japan from other countries within the last two weeks,” you will be ineligible for our screening program. Please visit a designated medical institution for infectious disease assigned by local government and get tested for SARS-CoV-2 infection.

**< About this screening >**

- This screening program will be performed at an external medical laboratory using the PCR kit approved by Japanese government, under the supervision of the Clinical and Translational Research Center and the Center for Preventive Medicine at Keio University Hospital.
- Please collect 1-2 ml of saliva sample according to the collection manual using the container within the kit.
- Please note the following when collecting saliva samples:
- If you are collecting saliva sample within 30 minutes of having a meal, please gently rinse your mouth with water.
- Please don’t gargle with mouthwash within 30 minutes before sample collection.
- If you find it difficult to collect enough volume of saliva, please try massaging lightly over your submandibular gland (under the chin).
- If saliva spills over the container, please wipe it with disinfectant such as ethanol.
- Please fill out the application and consent form and have it checked by the personnel in charge of the screening program before getting tested. The answers you write in the form may make you ineligible for the screening.
- This test is to confirm that the tested individual does not secrete enough virus to infect others, and not to exclude the possibility of SARS-CoV-2 infection. It is possible that anyone who gets tested here may later develop COVID-19 and have higher viral loads to infect others. If you start to feel unwell or notice changes in your health, please get re-tested at a designated hospital for infectious disease.
- Even if your screening test comes back as negative, you can get infected later. Please continue your daily infection prevention measures.
- If your screening test turns out to be positive, please follow the guidance as indicated in the manual. Our test is screening, and it is possible that even if you get a positive screening result, you may get a negative test result in the confirmatory diagnostic test.
- If you have any question regarding the system and content of this screening program, please reach out to the contact below.

Keio Cancer Center, Keio University School of Medicine

XXX, XXX, XXX

Tel: XXX, Email: XXX

**Application and Consent Form**

Please respond to the following questions.

Q1. Do you currently have flu-like symptoms, such as fever, coughing, and sore throat?

Yes No

(If yes, please describe your symptoms: )

Q2. Have you made a close contact with SARS-CoV-2 positive individuals in the last two weeks?

Yes No

Q3. Have you returned from abroad within the past two weeks?

Yes No

(If yes, please state the country (countries) you visited: )

Q4. This test is going to see if virus in saliva is detectable or not, but not exclude the possibility of current or future infection with SARS-CoV-2. Have you been informed about this, and do you understand what this means?

Yes No

Q5. The medical information we get from this screening (age, gender, test result, etc.) may be provided to medical research at Keio University School of Medicine (including so called “Donner Project”) or public health insurance administration. In such cases, your personal information will be protected. In addition, research will be conducted in accordance with ‘Ethical Guidelines for Medical and Health Research Involving Human Subjects’ mandated by the Ministry of Health, Labour and Welfare, Japan. Given the above information, will you agree that your medical information be used for these purposes?

Yes No

Based on the above, I give my consent to get PCR testing for SARS-CoV-2.

Date: XXXX/XXXX/XXXX

Branch: XXXXX

ID: XXXX

Name: XXXX

**If you receive a positive screening test result**

This screening test was designed to confirm that the tested individual does not secrete the amount of virus that might infect others, and is conducted in a different method and criterion from that used in the diagnostic PCR testing performed at medical facilities. It is possible that there may be false positive cases (in which the test returns as positive when it is actually negative). Therefore, the result from this screening alone does not confirm SARS-CoV-2 infection. Still, with the probability of SARS-CoV-2 positivity, please get an official COVID-19 diagnostic test at a nearby designated hospital for infectious disease assigned by local government in accordance with the following guides.

< Necessary actions>

- Minimize the possible risk to infect others.
- Avoid the three C’s (closed spaces, crowded places, and close-contact settings).

< Precautions to take before getting re-tested for COVID-19 >

- Avoid contact with others including family members, and be sure to wear a mask when you have to get in contact with them.
- Switch to remote working and refrain from going to your workplace.
- Eat meals alone and not with others including family members.
- If you are living with your family, do not share towels or bedclothes with your family, and wait until other family members finish taking a bath before you take a bath.
- Avoid using public transportation and use your car or your family member’s driving, and be sure to do enough ventilation if you have a company.

< If you have a negative test result in the re-test >

- You can go back to the same level of activity before the screening. You can report to work and use a public transportation with a mask.

< If you have a positive test result in the re-test >

- Please follow the instructions shared by the healthcare provider or the local public health center.
