# Supplementary material for: SARS-CoV-2 RT-qPCR testing of pooled saliva samples: A case study of 824 asymptomatic individuals and a questionnaire survey in Japan
Source: PLoS One. 2022 May 12;17(5):e0263700. doi: 10.1371/journal.pone.0263700 (PMC9098043; doi:10.1371/journal.pone.0263700)
Supplement: S2 File — (DOCX) [file pone.0263700.s006.docx]

Supporting information

*Pilot testing of pooling known positive and negative samples*

For initial evaluation, we used 25 saliva samples, determined as positive (4 samples) or negative (21 samples) by individual testing at Keio University Hospital. SARS-CoV-2 RNA in saliva was detected using a rapid RNA extraction-free RT-qPCR kit (SARS-CoV-2 Direct Detection RT-qPCR Kit; Takara Bio Inc., Shiga, Japan). This kit (approved by the Pharmaceuticals and Medical Devices Agency in Japan on October 27, 2020) (1) amplifies two regions of the nucleocapsid gene, N1 and N2, and a human internal control (IC) gene, as recommended by the US Centers for Disease Control and Prevention (2, 3).

Two pooling approaches were assessed: collecting and pooling before virus inactivation, or inactivating before pooling. In the former approach, ten 8-µl saliva samples were pooled in a microtube to total 80 μl, with 8 μl of the pooled sample mixed with 2 μl of sample preparation buffer (Solution A, included in the SARS-CoV-2 Direct Detection RT-qPCR Kit, Takara Bio Inc.), mixed by pipetting, incubated for 5 min at room temperature, and incubated at 95°C for 5 min. In the latter approach, inactivated saliva samples were combined in a microtube to total 20 μl. For a 10-sample pool, 10 samples of 2 μl each were collected; for a 20-sample pool, 20 samples of 1 μl each were collected. PCR was performed using a QuantStudio 5 Real-Time PCR System (Thermo Fisher Scientific, Waltham, MA, USA). The reaction mix contained 40 μl of reaction buffer, including the primers and probe, that was added to 10 μl of the prepared sample. Thermal cycling consisted of RT at 52°C for 5 min, 95°C for 10 s, followed by 45 cycles of denaturation at 95°C for 5 s and annealing/extension at 60°C for 30 s. A sample was considered as positive when amplification of a target region (N1 or N2 gene) was detected at a cycle threshold (Ct) of less than 40.

*Pilot testing result*

We used known positive and negative samples to produce two pools, including positive samples P1 and P2, pooled before virus inactivation. Each pool contained a single known positive sample and nine samples that had tested negative. P1 had a Ct of 26.4 when tested alone, and 30.7 when pooled. P2 had a Ct of 34 when tested alone, and 37.1 when pooled (Supplementary Table 1). Next, positive samples P3 and P4 were inactivated before being mixed with known negative samples. P3 had an individual Ct of 22.7 that increased to 26.1 in a 10-sample pool and to 28.2 in a 20-sample pool. P4 had an individual Ct of 34.8 that increased to 37.3 in a 10-sample pool and to 37.8 in a 20-sample pool (Supplementary Table 1). Taken together, all six pilot pools, each combining one known positive with nine or 19 known negative samples) tested positive. As expected, increased Ct values were observed following dilution.

1. Pharmaceuticals and Medical Devices Agency. PMDA’s Efforts to Combat COVID-19. Available from: <https://www.pmda.go.jp/english/about-pmda/0002.html> (accessed on Dec. 12, 2021).

2. Centers for Disease Control and Prevention. Research use only 2019-novel coronavirus (2019-nCcoVv) real-time RT-PCR primers and probes. Available from: <https://www.cdc.gov/coronavirus/2019-ncov/lab/rt-pcr-panel-primer-probes.html> (accessed on Aug. 10, 2021).

3. Zhen W, Berry GJ. Development of a New Multiplex Real-Time RT-PCR Assay for Severe Acute Respiratory Syndrome Coronavirus 2 (SARS-CoV-2) Detection. J Mol Diagn. 2020;22(12):1367-72.
