# Supplementary material for: SARS-CoV-2 RT-qPCR testing of pooled saliva samples: A case study of 824 asymptomatic individuals and a questionnaire survey in Japan
Source: PLoS One. 2022 May 12;17(5):e0263700. doi: 10.1371/journal.pone.0263700 (PMC9098043; doi:10.1371/journal.pone.0263700)
Supplement: Attachment — Submitted filename: PONE-D-22-02378_gtb.pdf [file pone.0263700.s007.pdf]

PLOS ONE

SARS-CoV-2 RT-qPCR testing of pooled saliva samples: a case study of 824 asymptomatic individuals and a questionnaire survey in Japan

--Manuscript Draft--

**Manuscript Number:**	PONE-D-22-02378
**Article Type:**	Research Article
**Full Title:**	SARS-CoV-2 RT-qPCR testing of pooled saliva samples: a case study of 824 asymptomatic individuals and a questionnaire survey in Japan
**Short Title:**	Pooled RT-qPCR testing of saliva samples for COVID-19 from asymptomatic individuals and a questionnaire survey
**Corresponding Author:**	Hiroaki Taniguchi, M.D., Ph.D.<br>Keio University - Shinanomachi Campus: Keio Gijuku Daigaku - Shinanomachi Campus<br>Shinjuku-ku, Tokyo JAPAN
**Keywords:**	COVID-19; RT-qPCR; Saliva; pooled testing; mass screening
**Abstract:**	From the beginning of the COVID-19 pandemic, the demand for diagnostic and screening tests has exceeded supply. Although the proportion of vaccinated people has increased in wealthier countries, breakthrough infections have occurred amid the emergence of new variants. Pooled-sample COVID-19 testing using saliva has been proposed as an efficient, inexpensive, and non-invasive method to allow larger-scale testing, especially in a screening setting. In this study, we aimed to evaluate pooled RT-qPCR saliva testing and to compare the results with individual tests.<br>Employees of Philips Japan, Ltd. were recruited to participate in COVID-19 screening from October to December 2020. Asymptomatic individuals (n=824) submitted self-collected saliva samples. Samples were tested for the presence of SARS-CoV-2 by RT-qPCR in both 10-sample pools and individual tests. We also surveyed participants regarding their thoughts and behaviors after the PCR screening project.<br>Two of the 824 individuals were positive by RT-qPCR. In the pooled testing, one of these two had no measurable Ct value, but showed an amplification trend at the end of the PCR cycle. Both positive individuals developed cold-like symptoms, but neither required hospitalization. Of the 824 participants, 471 responded to our online questionnaire. Overall, while respondents agreed that PCR screening should be performed regularly, the majority were willing to undergo PCR testing only when it was provided for free or at low cost.<br>In conclusion, pooled testing of saliva samples can support frequent large-scale screening that is rapid, efficient, and inexpensive.
**Order of Authors:**	Junna Oba, M.D., Ph.D.
	Hiroaki Taniguchi, M.D., Ph.D.
	Masae Sato
	Masaki Takanashi
	Moe Yokemura
	Yasunori Sato, M.D., Ph.D.
	Hiroshi Nishihara, M.D., Ph.D.
**Additional Information:**	
**Question**	**Response**
**Financial Disclosure**<br><br>Enter a financial disclosure statement that describes the sources of funding for the work included in this submission. Review	This work was partially supported by the Keio University Global Research Institute (KGRI) Research Projects for New Coronavirus Crisis: Implementation of a Keio Model to Optimize SARS-CoV-2 PCR Tests through Systems Approach (PI: Koichi Matsuo), the Japan Agency for Medical Research and Development (AMED) (PI: Hiroshi Nishihara, Grant Number 20he1422004j0001), Grant-in-Aid for Scientific Research (C)

Powered by Editorial Manager® and ProduXion Manager® from Aries Systems Corporation

the submission guidelines for detailed requirements. View published research articles from *PLOS ONE* for specific examples.<br><br>This statement is required for submission and **will appear in the published article** if the submission is accepted. Please make sure it is accurate.<br><br>**Unfunded studies**<br>Enter: *The author(s) received no specific funding for this work.*<br><br>**Funded studies**<br>Enter a statement with the following details:<br>• Initials of the authors who received each award<br>• Grant numbers awarded to each author<br>• The full name of each funder<br>• URL of each funder website<br>• Did the sponsors or funders play any role in the study design, data collection and analysis, decision to publish, or preparation of the manuscript?<br>• **NO** - Include this sentence at the end of your statement: *The funders had no role in study design, data collection and analysis, decision to publish, or preparation of the manuscript.*<br>• **YES** - Specify the role(s) played.<br><br>* typeset	of JSPS KAKENHI (PI: Junna Oba, Grant Number JP21K10334), and the Ministry of Education, Culture, Sports, Science and Technology of Japan (MEXT) for utilization of the university's PCR equipment. The funding agencies had no role in the study design, collection, analysis, or interpretation of data; in the writing of the report; or in the decision to submit the article for publication.
**Competing Interests**<br><br>Use the instructions below to enter a competing interest statement for this submission. On behalf of all authors, disclose any competing interests that could be perceived to bias this work—acknowledging all financial support and any other relevant financial or non-financial competing interests.<br><br>This statement is required for submission and **will appear in the published article** if the submission is accepted. Please make sure it is accurate and that any funding sources listed in your Funding Information later in the submission form are also	The authors have declared that no competing interests exist.

declared in your Financial Disclosure statement.<br><br>View published research articles from *PLOS ONE* for specific examples.<br><br>**NO authors have competing interests**<br><br>Enter: *The authors have declared that no competing interests exist.*<br><br>**Authors with competing interests**<br><br>Enter competing interest details beginning with this statement:<br><br>*I have read the journal's policy and the authors of this manuscript have the following competing interests: [insert competing interests here]*<br><br>* typeset	
**Ethics Statement**<br><br>Enter an ethics statement for this submission. This statement is required if the study involved:<br><br>• Human participants<br>• Human specimens or tissue<br>• Vertebrate animals or cephalopods<br>• Vertebrate embryos or tissues<br>• Field research<br><br>Write "N/A" if the submission does not require an ethics statement.<br><br>General guidance is provided below. Consult the submission guidelines for detailed instructions. **Make sure that all information entered here is included in the Methods section of the manuscript.**	This study was approved by the Institutional Review Boards at Keio University (IRB approval number 20200291). The study was conducted according to the principles expressed in the Declaration of Helsinki of 1964 and later versions. Written informed consent was obtained from all individuals who participated in the PCR screening tests, and online informed consent was obtained from all individuals who participated in the questionnaire survey, all prior to data collection.

**Format for specific study types**<br><br>**Human Subject Research (involving human participants and/or tissue)**<br>• Give the name of the institutional review board or ethics committee that approved the study<br>• Include the approval number and/or a statement indicating approval of this research<br>• Indicate the form of consent obtained (written/oral) or the reason that consent was not obtained (e.g. the data were analyzed anonymously)<br><br>**Animal Research (involving vertebrate animals, embryos or tissues)**<br>• Provide the name of the Institutional Animal Care and Use Committee (IACUC) or other relevant ethics board that reviewed the study protocol, and indicate whether they approved this research or granted a formal waiver of ethical approval<br>• Include an approval number if one was obtained<br>• If the study involved *non-human primates*, add *additional details* about animal welfare and steps taken to ameliorate suffering<br>• If anesthesia, euthanasia, or any kind of animal sacrifice is part of the study, include briefly which substances and/or methods were applied<br><br>**Field Research**<br><br>Include the following details if this study involves the collection of plant, animal, or other materials from a natural setting:<br>• Field permit number<br>• Name of the institution or relevant body that granted permission	
**Data Availability**<br><br>Authors are required to make all data underlying the findings described fully available, without restriction, and from the time of publication. PLOS allows rare exceptions to address legal and ethical concerns. See the PLOS Data Policy and FAQ for detailed information.	Yes - all data are fully available without restriction

Powered by Editorial Manager® and ProduXion Manager® from Aries Systems Corporation

A Data Availability Statement describing where the data can be found is required at submission. Your answers to this question constitute the Data Availability Statement and **will be published in the article**, if accepted.<br><br>**Important:** Stating 'data available on request from the author' is not sufficient. If your data are only available upon request, select 'No' for the first question and explain your exceptional situation in the text box.<br><br>Do the authors confirm that all data underlying the findings described in their manuscript are fully available without restriction?	
**Describe where the data may be found in full sentences. If you are copying our sample text, replace any instances of XXX with the appropriate details.**<br><br>• If the data are **held or will be held in a public repository**, include URLs, accession numbers or DOIs. If this information will only be available after acceptance, indicate this by ticking the box below. For example: *All XXX files are available from the XXX database (accession number(s) XXX, XXX.).*<br>• If the data are all contained **within the manuscript and/or Supporting Information files**, enter the following: *All relevant data are within the manuscript and its Supporting Information files.*<br>• If neither of these applies but you are able to provide **details of access elsewhere**, with or without limitations, please do so. For example:<br><br>*Data cannot be shared publicly because of [XXX]. Data are available from the XXX Institutional Data Access / Ethics Committee (contact via XXX) for researchers who meet the criteria for access to confidential data.*<br><br>*The data underlying the results presented in the study are available from (include the name of the third party*	All relevant data are within the manuscript and its Supporting Information files.

and contact information or URL).<br>• This text is appropriate if the data are owned by a third party and authors do not have permission to share the data.<br><br>* typeset	
Additional data availability information:	

Manuscript

Click here to access/download;Manuscript;Pooled_testing_Manuscript_2022

SARS-CoV-2 RT-qPCR testing of pooled saliva samples: a case study of 824 asymptomatic individuals and a questionnaire survey in Japan

Junna Oba[1,2], Hiroaki Taniguchi[1,3,4*], Masae Sato[1], Masaki Takanashi[5], Moe Yokemura[5], Yasunori Sato[6], Hiroshi Nishihara[1,3,4]

[1] Keio Cancer Center, Keio University School of Medicine, 35 Shinanomachi, Shinjuku-ku, Tokyo 160-8582, Japan

[2] Department of Extended Intelligence for Medicine, The Ishii-Ishibashi Laboratory, Keio University School of Medicine, 35 Shinanomachi, Shinjuku-ku, Tokyo 160-8582, Japan

[3] Research and Development Center for Precision Medicine, University of Tsukuba, Innovation Medical Research Institute, 1-2 Kasuga, Tsukuba-shi, Ibaraki 305-8550, Japan

[4] Keio University Hospital Clinical and Translational Research Center, Keio University School of Medicine, 35 Shinanomachi, Shinjuku-ku, Tokyo 160-8582, Japan

[5] LSI Medience Corporation Central Laboratory Center, 3-30-1 Shimura, Itabashi-ku, Tokyo 174-8555, Japan

[6] Department of Preventive Medicine and Public Health, Keio University School of Medicine, 35 Shinanomachi, Shinjuku-ku, Tokyo 160-8582, Japan

* Correspondence: h-tani@keio.jp; Tel. +81-3-5315-4375

Abstract

From the beginning of the COVID-19 pandemic, the demand for diagnostic and screening tests has exceeded supply. Although the proportion of vaccinated people has increased in wealthier countries, breakthrough infections have occurred amid the emergence of new variants. Pooled-sample COVID-19 testing using saliva has been proposed as an efficient, inexpensive, and non-invasive method to allow larger-scale testing, especially in a screening setting. In this study, we aimed to evaluate pooled RT-qPCR saliva testing and to compare the results with individual tests.

Employees of Philips Japan, Ltd. were recruited to participate in COVID-19 screening from October to December 2020. Asymptomatic individuals (n=824) submitted self-collected saliva samples. Samples were tested for the presence of SARS-CoV-2 by RT-qPCR in both 10-sample pools and individual tests. We also surveyed participants regarding their thoughts and behaviors after the PCR screening project.

Two of the 824 individuals were positive by RT-qPCR. In the pooled testing, one of these two had no measurable Ct value, but showed an amplification trend at the end of the PCR cycle. Both positive individuals developed cold-like symptoms, but neither required hospitalization. Of the 824 participants, 471 responded to our online questionnaire. Overall, while respondents agreed that PCR screening should be performed regularly, the majority were willing to undergo PCR testing only when it was provided for free or at low cost.

In conclusion, pooled testing of saliva samples can support frequent large-scale screening that is rapid, efficient, and inexpensive.

Keywords

COVID-19, RT-qPCR, saliva, pooled testing, mass screening

Introduction

The COVID-19 pandemic has altered our daily lives. Although vaccination programs have progressed in many countries, daily infections and hospitalizations are still high globally (1, 2). Moreover, breakthrough infections in fully vaccinated individuals have been reported, indicating that such people can still contract and transmit the virus (3-5). One of the challenges in controlling this disease is that the spread of SARS-CoV-2 infection occurs not only in symptomatic patients but also in asymptomatic carriers, including many who later develop symptoms (presymptomatic cases) (6-8). Further, as community transmission continues, new variants are likely to emerge; when we drafted this manuscript, the highly transmissible Delta variant had replaced the majority of strains worldwide, but started to be replaced by a newer variant Omicron (9).

Early identification and isolation of infected individuals through comprehensive screening is effective in minimizing the spread of infection. The gold standard diagnostic test for SARS-CoV-2 infection is real-time reverse transcription – polymerase chain reaction (RT-qPCR) using upper respiratory tract specimens (10). While nasopharyngeal swabs (NPS) have been the most widely used sample type, collecting NPS samples requires trained personnel and personal protective equipment (PPE), increasing infection risks to both patients and healthcare workers; moreover, it causes discomfort and a potential risk of complications to patients because of the anatomical structures of the nasal cavity (11-13). These factors have limited the widespread use of NPSs.

Using saliva samples is an attractive alternative. It has advantages over NPS samples: it is much less invasive and allows self-collection, making it more feasible for repeated, frequent testing; it minimizes the infection risk during sample collection; and has lower personnel and PPE costs (14). Some studies that compared the sensitivity and specificity of SARS-CoV-2 diagnostic RT-qPCR tests between saliva and NPS samples have shown that sensitivity is higher using NPS (15-17), while others show that saliva samples have a higher (14, 18, 19) or similar (20-22) sensitivity. Recent meta-analyses have shown that there is little difference in sensitivity between NPS and saliva samples for the detection of SARS-CoV-2 nucleic acid (23-25). Although multiple studies have reported the presence of viral RNA

in saliva samples from both symptomatic and asymptomatic patients (21, 26, 27), the timing of sample collection seems to be important, because the viral load of SARS-CoV-2 in saliva declines after disease onset (18, 28, 29), highlighting the importance of collecting samples during the early phase of disease. Thus, saliva sampling is not only easier, safer, and less expensive, but also a reliable option for COVID-19 testing.

Pooled testing, in which samples from multiple individuals are combined into a single test, havs been shown to be effective in multiple infectious disease screening settings, including for syphilis and HIV (30, 31). During the current COVID-19 pandemic, a pooled-sample testing approach has been reported to save both cost and time when implemented on larger scales (30, 32, 33). For pooled testing to be effective, certain baseline parameters should be considered: the prevalence or positivity rate within the community, sensitivity and specificity of the test, and the limits of detection. Multiple studies have published models and algorithms to calculate optimal pool sizes that depend on prevalence and cost reductions relative to individual tests (34-38). Overall, sample pooling is most advantageous for populations with low prevalence, remaining more time- and cost-efficient than individual testing in populations with positivity rates up to around 30%; however, it offers no benefit when the positivity rate (prevalence) becomes higher (34-38).

In our cohort of healthy and asymptomatic individuals who were scheduled for medical check-ups at the Center for Preventive Medicine at Keio University Hospital, only 3 of 2,342 (0.19%) individuals tested between August and December 2020 had positive results (39), indicating low prevalence among asymptomatic individuals during this period. Taken together, pooled testing of saliva samples, especially for screening in communities with relatively low prevalence, should allow more efficient utilization of resources and more rapid screening of a greater number of people with faster turnaround times.

We proposed the concept of social PCR testing, which allows safe social and economic activities by routine testing of saliva samples from asymptomatic individuals (39). Parallel implementation of mass screening coupled with sample pooling has been reported to contribute to the

success of COVID-19 control (40-42). Here, we report the results of SARS-CoV-2 PCR screening tests performed on 824 employees of Philips Japan, Ltd., who had neither symptoms nor close contact with infected individuals during the period of October through December 2020. Further, we analyzed the results of web questionnaires completed by 471 employees who had participated in the PCR screening regarding their attitudes toward mass-screening policies.

Materials and Methods

Pilot testing of pooling known positive and negative samples

For initial evaluation, we used 25 saliva samples, determined as positive (4 samples) or negative (21 samples) by individual testing at Keio University Hospital. SARS-CoV-2 RNA in saliva was detected using a rapid RNA extraction-free RT-qPCR kit (SARS-CoV-2 Direct Detection RT-qPCR Kit; Takara Bio Inc., Shiga, Japan). This kit (approved by the Pharmaceuticals and Medical Devices Agency in Japan on October 27, 2020) (43) amplifies two regions of the nucleocapsid gene, N1 and N2, and a human internal control (IC) gene, as recommended by the US Centers for Disease Control and Prevention (44, 45).

Two pooling approaches were assessed: collecting and pooling before virus inactivation, or inactivating before pooling. In the former approach, ten 8-µl saliva samples were pooled in a microtube to total 80 µl, with 8 µl of the pooled sample mixed with 2 µl of sample preparation buffer (Solution A), mixed by pipetting, incubated for 5 min at room temperature, and incubated at 95°C for 5 min. In the latter approach, inactivated saliva samples were combined in a microtube to total 20 µl. For a 10-sample pool, 10 samples of 2 µl each were collected; for a 20-sample pool, 20 samples of 1 µl each were collected. PCR was performed using a QuantStudio 5 Real-Time PCR System (Thermo Fisher Scientific, Waltham, MA, USA). The reaction mix contained 40 µl of reaction buffer, including the primers and probe, that was added to 10 µl of the prepared sample. Thermal cycling consisted of RT at 52°C for 5 min, 95°C for 10 s, followed by 45 cycles of denaturation at 95°C for 5 s and annealing/extension at 60°C for 30 s. A sample was considered as positive when amplification of a target region (N1 or N2 gene) was detected at a cycle threshold (Ct) of less than 40.

Testing of unknown samples

Between October and December 2020, employees of Philips Japan, Ltd. were asked to participate in RT-qPCR screening. A total of 824 volunteers who provided informed consent were enrolled in this study. Eligible individuals included those who did not report fever above 37.5° C or

coughing, and had not been in close contact with COVID-19 patients during the two weeks preceding the test. Participants were instructed to collect 1–2 ml of saliva using a FastGene™ Saliva Collection Kit (Nippon Genetics Co., Ltd., Tokyo, Japan), at home, without restrictions on food timing or intake. Samples were collected at each branch office, kept at 4°C, and sent to the LSI Medience Corporation Central Laboratory (Itabashi-ku, Tokyo, Japan), where all PCR testing was performed within 24 h of receipt. For both test modes, samples were vortexed for 5 s and centrifuged at 2500 rpm for 3 min. For pooled testing, equal volumes of saliva supernatant (20 μl each) from 10 subjects were pooled. When the number of samples in a pool was less than 10, phosphate buffered saline was added to bring the volume to 200 μl. Each individual or pooled 200-ul sample was mixed with 200 μl of lysis buffer and 20 μl of proteinase K solution for inactivation, followed by vortexing, heating for 10 min at 56°C, and brief centrifugation. RNA was extracted using the Maxwell RSC 48 Instrument and Viral Total Nucleic Acid Purification Kit (Promega, Madison, WI, USA) and eluted into a total volume of 50 μl. For RT-qPCR, 10 μl of RNA template was mixed with 40 μl of PCR Master Mix and amplified using a SARS-CoV-2 Detection Kit -Multi- (TOYOBO Co., Ltd., Osaka, Japan) targeting the N1 and N2 genes, as well as an IC. This detection kit was developed for research use, but was approved for clinical use by the Japanese government in August 2020. RT-qPCR was performed using the COBAS z480 instrument (Roche Diagnostics K.K., Tokyo, Japan). The kit protocol was followed: 42°C for 5 min for RT; pre-denaturation at 95°C for 10 s; and 45 cycles of 95°C for 5 s and 60°C for 30 s for amplification. Ct values were determined for N1, N2, and IC. Samples were considered positive for SARS-CoV-2 when either the N1 or N2 target was detected with a Ct < 40.

Online survey

The 824 subjects who participated in the PCR screening were asked to respond anonymously to an online survey on how they felt about the social PCR screening between May 19 and May 26, 2021. Participants received a clear explanation of the survey procedure and could interrupt or terminate the survey at any time without giving a reason.

Statistical Analysis

For the questionnaire, sex differences in demographic characteristics and responses were assessed using Pearson’s chi-squared test or Fisher’s exact test, where appropriate. Statistical significance was set at $p < 0.05$. Data visualization and statistical analyses were performed using R v4.1.1.

Ethics statement

This study was approved by the institutional review board of Keio University (approval number 20200291). The study was conducted according to the principles of the Declaration of Helsinki of 1964 and later versions. Written informed consent was obtained from all individuals who participated in the PCR screening tests, and informed consent was obtained from all individuals who participated in the survey, all prior to data collection.

Results

Pilot testing

We used known positive and negative samples to produce two pools, including positive samples P1 and P2, pooled before virus inactivation. Each pool contained a single known positive sample and nine samples that had tested negative. P1 had a Ct of 26.4 when tested alone, and 30.7 when pooled. P2 had a Ct of 34 when tested alone, and 37.1 when pooled (Supplementary Table 1). Next, positive samples P3 and P4 were inactivated before being mixed with known negative samples. P3 had an individual Ct of 22.7 that increased to 26.1 in a 10-sample pool and to 28.2 in a 20-sample pool. P4 had an individual Ct of 34.8 that increased to 37.3 in a 10-sample pool and to 37.8 in a 20-sample pool (Supplementary Table 1).

Taken together, all six pilot pools, each combining one known positive with nine or 19 known negative samples) tested positive. As expected, increased Ct values were observed following dilution.

Pooled versus individual testing of 1060 samples

Saliva samples from 824 subjects without symptoms or exposure in the preceding two weeks were tested in 10-sample pools and individually. Of the 824 individuals, 212 submitted two samples at different times during the study period; six submitted three times, one submitted four times, one submitted five times, and one submitted six times. The remaining 603 submitted samples once. Two of the 1060 samples (two of the 824 individuals) were positive. In case 1, 10-sample pool testing detected the N1 signal (Ct=33.71) but did not detect N2, while individual testing detected both N1 and N2 (Ct 33.84 and 35.82, respectively) (Figure 1A, Table 1). In case 2, neither N1 nor N2 signals were detected in pooled testing, whereas individual testing identified both N1 and N2 (Ct 31.81 and 33.24, respectively) (Figure 1B, Table 2). In both cases, individual testing showed sigmoid amplification curves for N1 and N2 at Ct values between 30 and 35. In case 2, the 10-sample pool exhibited no Ct value, ~~but showed the start of a sigmoid amplification curve at the end of the cycle (red dotted box in Figure 1B).~~ However, neither of the cases had Ct values or showed any late amplification of N2 in pools.

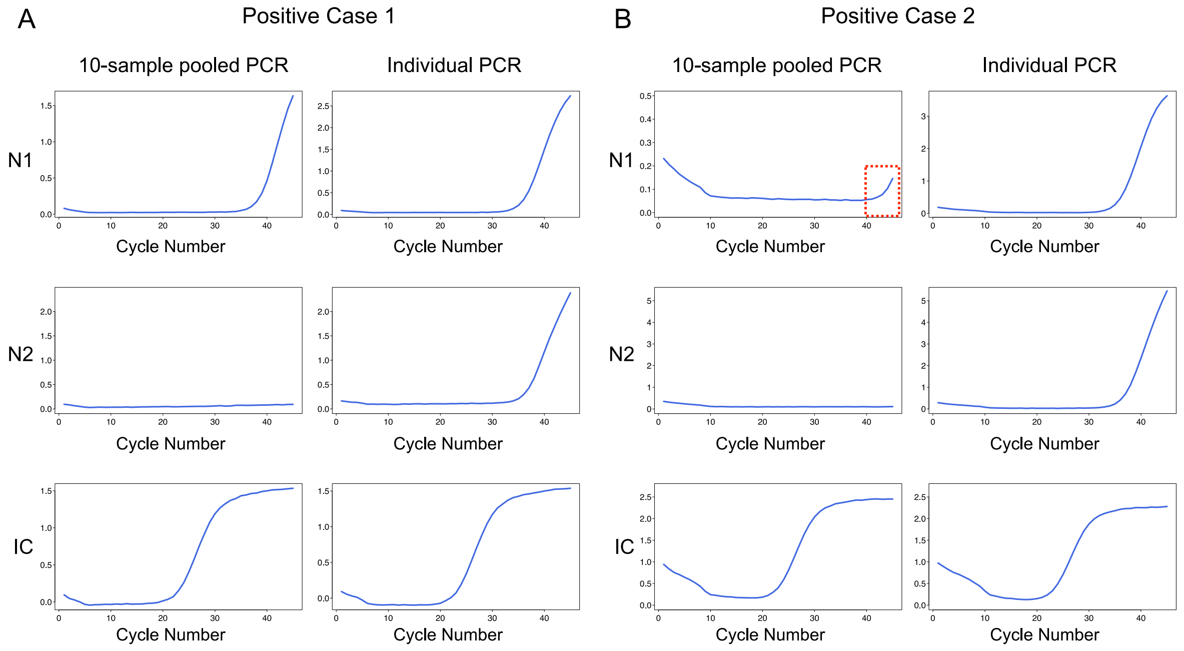

Figure 1. PCR amplification curves for positive case 1 (A) and positive case 2 (B). Upper panel, N1; middle panel, N2; lower panel, human internal control (IC) gene. Left column of each panel, 10-sample pools; right column of each panel, individual samples.

Table 1. Individual amplification of 10 samples from the pool including positive case 1 (#8).

**Sample Name**	**Result**		**Ct (N1)**		**Ct (N2)**		**Ct (IC)**	
	**Individual**	**Pool**	**Individual**	**Pool**	**Individual**	**Pool**	**Individual**	**Pool**
#1	Not detected	Probably positive	-	33.71	-	-	22.76	22.81
#2	Not detected		-		-		22.88	
#3	Not detected		-		-		22.80	
#4	Not detected		-		-		22.72	
#5	Not detected		-		-		22.85	
#6	Not detected		-		-		23.13	
#7	Not detected		-		-		22.73	
#8	Positive		33.84		35.82		22.84	
#9	Not detected		-		-		22.80	
#10	Not detected		-		-		22.73	

When the Ct value for either N1 or N2 was less than 40, samples were scored as positive for individual samples and probably positive for 10-sample pools; for unmeasurable Ct values shown as dashes, the both test modes were scored as not detected.

Table 2. Individual amplification of 10 samples from the pool including positive case 2 (#20).

**Sample Name**	**Result**		**Ct (N1)**		**Ct (N2)**		**Ct (IC)**	
	**Individual**	**Pool**	**Individual**	**Pool**	**Individual**	**Pool**	**Individual**	**Pool**
#11	Not detected	Not detected	-	-	-	-	26.58	29.67
#12	Not detected		-		-		28.31	

#13	Not detected		-		-		26.94	
#14	Not detected		-		-		27.10	
#15	Not detected		-		-		29.88	
#16	Not detected		-		-		28.05	
#17	Not detected		-		-		28.68	
#18	Not detected		-		-		32.97	
#19	Not detected		-		-		27.26	
#20	Positive		31.81		33.24		28.67	

Samples were scored as positive when either N1 or N2 was detected with a Ct value of less than 40; samples were scored as not detected when Ct values were unmeasurable, shown as dashes.

Screening summary and PCR amplification inhibition

Overall, for 1060 samples, 110 pooled- and 1060 individual tests were performed (Table 3). Only one pool was scored as probably positive; individual tests confirmed one positive case from this pool. PCR amplification was sometimes inhibited, as indicated by increased Ct for the IC genes. In general, the IC Ct values in most samples were 20-30. We defined inhibition when an IC Ct was unmeasurable or above 40, or when we observed no sigmoid amplification pattern (Figure 2). In total, 74 individual samples and one pooled sample were in this category (Table 3). Interestingly, these 74 samples showed normal range IC Ct values in pooled testing. These results indicate that pooling may have mitigated the inhibition of amplification observed in individual tests.

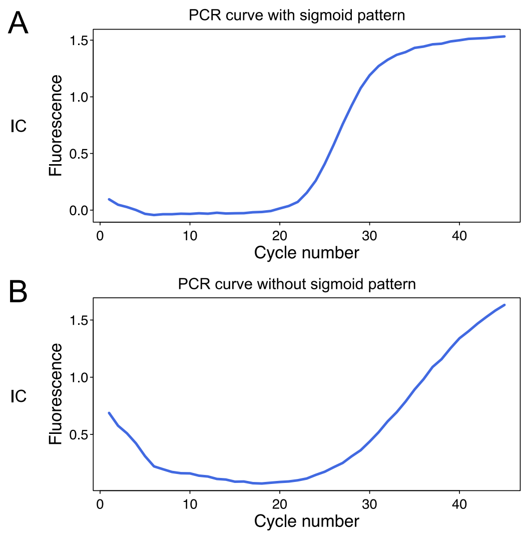

Figure 2. Examples of good and poor PCR amplification. (A) Good amplification with a sigmoid pattern. (B) Poor amplification lacking a sigmoid pattern.

Table 3. Pooled and individual testing.

Date of sample receipt	Number of samples tested	10-sample pool PCR				Individual PCR		
		Number of pools	Probably positive	Not detected	Amplification inhibition	Positive	Not detected	Amplification inhibition
2020/10/22	87	9	0	9	0	0	87	0
2020/11/05	101	11	1	10	0	1	97	3
2020/11/13	272	28	0	28	0	0	268	4
2020/11/19	41	5	0	4*	1	1	39	1
2020/11/26	88	9	0	9	0	0	87	1
2020/12/03	137	14	0	14	0	0	136	1
2020/12/10	130	13	0	13	0	0	117	13
2020/12/11	70	7	0	7	0	0	60	10
2020/12/17	134	14	0	14	0	0	93	41
Total	1060	110	1	108	1	2	984	74

*There was a sharp amplification curve for N1 at Ct higher than 40 in one pool.

Clinical courses of the two positive cases

Case one was had felt chills six days prior to sample collection and visited the local clinic the next day, presenting symptoms including nasal discharge, sore throat, and sputum. She was diagnosed as having a common cold, not tested for SARS-CoV-2, and was prescribed medicine. She still had cold-like symptoms without fever when she submitted her sample on November 4, 2020. On receiving the positive PCR test result, she underwent another PCR test with a saliva sample at a clinic two days after our screening, which was negative. She had no history of travel or exposure. She self-quarantined and monitored her symptoms for two weeks. She had mild cold-like symptoms for 9-10 days, but developed no fever and required no treatment.

Case two had no symptom when he submitted his sample on November 18, 2020. However, he started to cough the next evening when he received the positive test result, and developed more serious symptoms in the next morning, including fatigue, chills, fever (38.9 °C), headache, joint pain, sore throat, and severe coughing. He tested positive at a local clinic two days after the initial test. When questioned regarding possible exposure, he revealed that he had attended a wedding reception three days prior to the initial sampling, and took off his mask while eating the meal. The fever subsided within two days, but symptoms of fatigue, coughing, sore throat, diarrhea, and altered smell and taste continued for 6-7 days. These symptoms gradually resolved and he required no medical intervention during two weeks of home quarantine.

Questionnaire

Of the 824 individuals who participated in PCR screening test, 476 (57.8%) completed the online questionnaire. As five respondents indicated that they did not want their survey results to be used for research purposes, we analyzed the responses from the remaining 471. The respondents included 358 males and 113 females, the majority between the ages of 30 and 50 years (Figure 3A). The locations of the participants were distributed across Japan, with the top three sites in the Kanto area (Tokyo, Kanagawa, and Saitama) (Figure 3B).

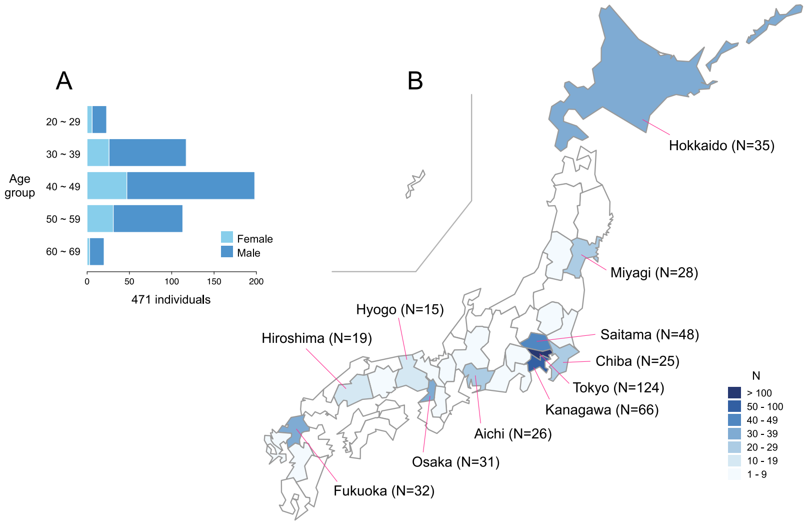

Figure 3. Characteristics of survey participants. (A) Age group distribution with males (blue) and females (light blue). (B) Locations at the time of screening, showing prefectures with more than 10 participants.

Although no state of emergency was declared by the government during the study period, 62.8% of the respondents did not travel in the two weeks before testing (Q1, Figure 4). Most (79.8%) reported that they felt relieved by seeing the result (Q2). The 132 who responded "yes" to Q4 (Has your awareness been changed after participating in this screening project?) (N=132) were subsequently asked to provide more details, and 131 individuals gave analyzable comments: 81 commented on their awareness of infection prevention, stating that they became more careful or felt assured by their preventative measures (washing hands, avoiding crowds, limiting unnecessary outings, etc.); 42 commented on PCR testing: 23 noted that they realized that saliva PCR testing was easy and helpful; and 19 stated that the PCR testing should be performed regularly. The remaining eight commented that the relief of a negative test result is temporary and that PCR is not 100% accurate (Figure 4).

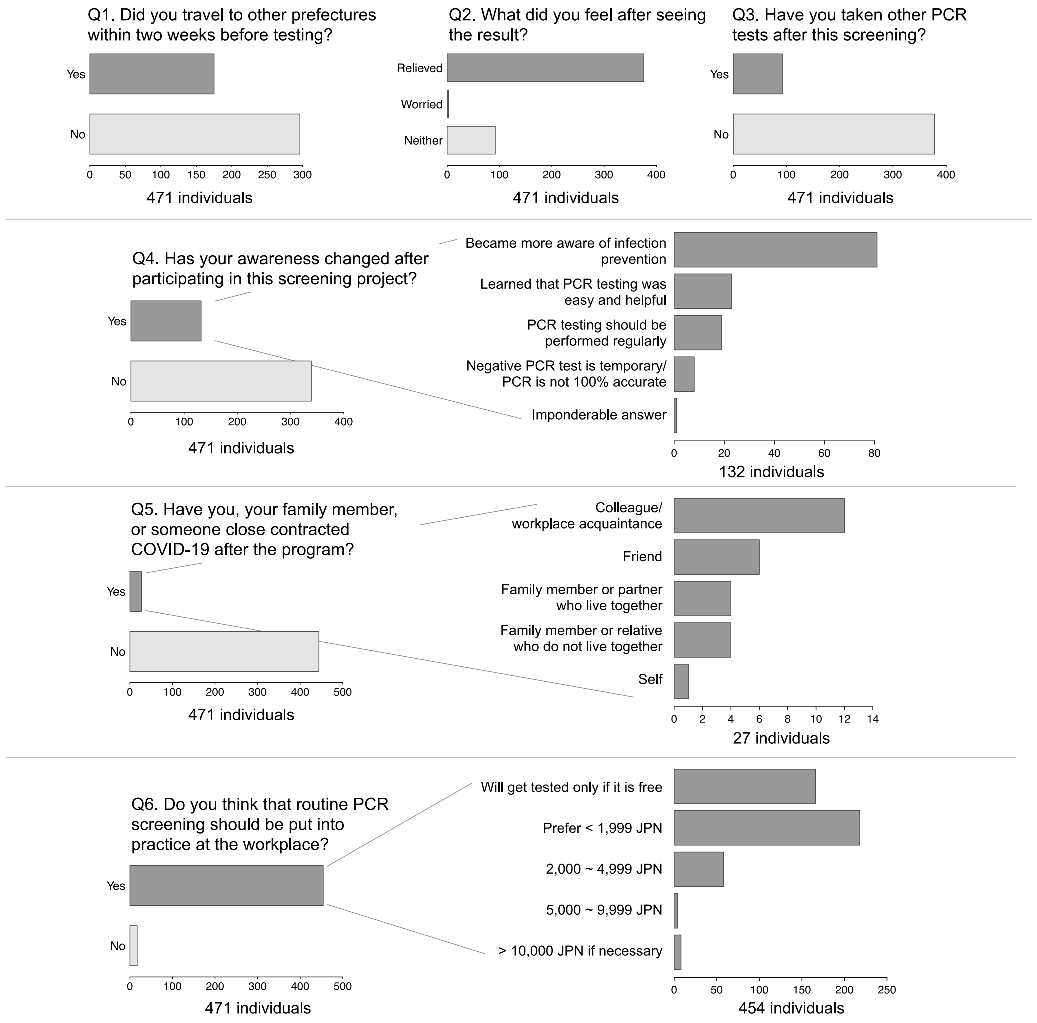

Figure 4. Responses to the online survey.

Individuals who answered “yes” to Q5 (Have you, your family member, or someone close contracted COVID-19 after the screening program?) (N=27) subsequently gave more specifics on who had contracted COVID-19: one reported his/her own infection, four reported that family members or living partners had become infected, and four reported that relatives who did not live with them later tested positive, six reported friends, and twelve reported colleagues or acquaintances at their workplace (Figure 4). Those who responded “yes” to Q6 (Do you think that routine PCR screening should be put into practice at the workplace?) were subsequently asked how much they would be willing to pay for such testing. It is notable that while the overwhelming majority of respondents agreed that routine PCR screening should be put into practice at the workplace, many were unwilling to pay out-of-pocket costs;

166 (38.6%) answered that they would not be tested unless the workplace tests were free, and 218 (48%) answered that they would pay if the cost was less than 1,999 JPN (approximately US$20) (Figure 4). Demographic characteristics of the responders, questionnaire responses, and statistical tests for gender differences are summarized in Supplementary Table 2. Statistically significant difference between sexes were seen only for location (Fisher's exact test, p = 0.0005), Q3 (if participants underwent other PCR tests after this program, Chi-squared test p = 0.0078), and Q4-1 (specificity of awareness change, Fisher's exact test p = 0.036).

Discussion

Although SARS-CoV-2 vaccines reduce the risk of symptomatic and severe disease and infection (46, 47), breakthrough infections occur (3-5) and vaccine effectiveness against transmission is decreased for the delta variants (48, 49). Additionally, vaccine protective effects decline considerably after six months, especially in older people, according to studies in the UK (50) and Israel (51). The recent surge of infections with delta and omicron variants across the world demonstrates that vaccines alone cannot fully contain viral spread, and that efficacy decreases over time and with immune escape acquired by viral mutations (52-55). It is important to keep in mind that vaccines alone cannot end the pandemic; therefore, we need to orchestrate all available tools to win the fight against this global scourge, including rapid testing and screening, physical distancing, and sanitary precautions (56). Pooled testing has been shown to be a reliable option for rapid, large-scale, and cost-effective screening.

In this study, we compared the RT-qPCR results of pooled and individual saliva samples from 824 subjects, and reported a follow-up questionnaire survey of 471 of them. Of the 1060 total samples, only those from two individuals were positive (2 of 824 individuals, 0.24%). The positive rate in this cohort was similar to that observed in another cohort of healthy/asymptomatic individuals who underwent RT-qPCR screening before medical checkups at the Center for Preventive Medicine at Keio University Hospital during the same period (39). To compare and contrast our data with official public

health surveillance data, we analyzed 1) total numbers of PCR tests and positive cases for suspicious individuals/already diagnosed patients during the same period from the Japan Ministry of Health, Labour and Welfare (https://covid19.mhlw.go.jp/en/) (Supplementary table 3); and 2) monitoring initiatives targeting asymptomatic individuals in 14 prefectures (Hokkaido, Miyagi, Tochigi, Saitama, Chiba, Tokyo, Kanagawa, Aichi, Gifu, Kyoto, Osaka, Hyogo, Fukuoka, and Okinawa) run by the Office for Novel Coronavirus Disease Control, Cabinet Secretariat, Government of Japan (https://corona.go.jp/monitoring/) in early 2021. In the first case (Supplementary Table 3), the mean positivity [(daily cases/tests) × 100] during the period was 13.3%, much higher than that in our cohort. This is probably because of the difference in the target populations; in the first case, PCR tests were performed for suspected individuals with symptoms or recent exposure or contact with confirmed COVID-19 patients. In the second, we see regional and time frame heterogeneity in positive rates, but rates ranged from 0 to 1.34%, comparable to our results.

Because pooling samples will dilute the viral load of a positive sample and tend to produce higher Ct, detecting samples with lower viral loads has been a major challenge in pooling approaches. In our study, Positive Case 2 was not detected in a 10-sample pool. The Ct values for N1 and N2 in the individual test for this case were 31.81 and 33.24, respectively. In our preliminary pooled-sample testing, 10-sample pools resulted in Ct increases ranging from 2.5 to 4.3 (median, 3.25). ~~If we simply add these values, Case 2 could have been detected in the 10-sample pool testing as well, with estimated Ct of 34.3~36.1 for N1 and 35.74~37.54 for N2, respectively. However, different RT-qPCR kits were used for the pilot tests (Takara) and the screening (Toyobo), so we cannot directly compare their results.~~ Consistent with our findings, previous studies showed similar Ct increases in pooled testing relative to individual testing, with variations in added values in pooled testing (15, 34, 57, 58). Interestingly, some groups observed no Ct differences between pooled and individual RT-qPCR testing (59, 60). More et al. reported that while individual positive samples with high viral load (Ct < 28) were consistently detected in pools of 5 or 10 with other clinical samples, there was a higher frequency of false negatives when samples with lower viral loads (Ct >28) were pooled, especially in pools of 10. They showed that

samples with individual Ct values >31 were not detected in pools of 10, whereas Ct values up to 33 could be detected in a pool of 5; they concluded that pooling up to five samples is more reliable for diagnostic purposes (61). Praharaj et al. compared 5- and 10-sample pooling and showed that the former had higher concordance with individual testing and lower false-negative rates than the latter; they also showed that 10-sample pools had lower concordance with individual-sample testing, and higher false-negative rates at Ct values of more than 30 cycles (62). In addition, Griesemer et al. reported that samples with Ct values of 33 or higher were detected in 95% of five-sample pools but only in 87% of nine-sample pools (63). Furthermore, Watkins et al. reported that sensitivity decreased with increasing pool size: pools of 5, 10, and 20 had reductions of 7.4, 11.1, and 14.8%, respectively (34). In both of the positive cases in our study, Ct values in individual testing were higher than 30, and 10-sample pools failed to produce any Ct values in case 2. Given the decrease in sensitivity in 10-sample pools, and with the two individual positive cases having high Ct values, it is probable that this case would have been undetected in a 10-sample pool if we had not performed simultaneous individual testing.

Mohanty et al. showed that considering late amplification while interpreting the results of pooled samples allowed the identification of additional positives (64). They first used criteria for positivity (Ct within 35 with a sigmoid curve) for 4-sample pools, but also included an additional class of 'probably positive' (Ct >35 with non-sigmoid amplification curve, or increased amplification at the end of the reaction). Adding this lenient cut-off yielded 15.5% more true-positive samples. Their study highlighted the importance of catching late amplification to avoid missing positive samples. When carefully reviewing the PCR curves from pooled testing for case 2, we observed late amplification patterns at the end of the reaction for N1, as described by Mohanty et al. (64).

We also encountered a relatively high frequency of PCR inhibition in individual tests, defined as Ct values of IC being unmeasurable or above 40, or by no sigmoid PCR amplification pattern. Saliva can contain inhibitors that impair nucleic acid amplification (65, 66). In addition to its molecular composition hindering RNA detection, saliva can be a challenging clinical sample because it varies

across individuals in pH and viscosity, as well as being more susceptible to the effects of RNases (65). Several protocols have been proposed to overcome these challenges, such as dilution, chemical pretreatment, heating, and treatment with proteinase K (66). In our study, pooled sample testing exhibited less amplification inhibition than individual testing, suggesting that pooling may reduce saliva's inhibitory effects on PCR.

As case 1 had cold-like symptoms six days prior to sample collection, strictly speaking, she was not asymptomatic. When she underwent another RT-qPCR test at the local clinic, it was eight days after the onset of symptoms, at a relatively late phase of infection. We speculate that this is the reason the test was negative. Case 2 had no symptoms when he submitted his samples, but had attended a wedding reception three days before. It was very likely that he was infected at the wedding and was presymptomatic (in an incubation period) when he participated in the PCR screening. The RT-qPCR test detected infection before he started to manifest symptoms. This finding reinforces the usefulness and clinical applicability of PCR testing.

In our study, several participants submitted samples more than once during the study period. There has been increasing evidence that success in containing SARS-CoV-2 depends more on the frequency and turnaround time of the testing than on the test being highly sensitive (67, 68).
Multiple factors can account for a high Ct (i.e., a weakly positive case): in very early phases of infection, where viral loads will likely increase, or during recovery, when fragmented viral RNA may be detected but may not be infective. In pooled testing, we may sometimes have negative false results because of decreased sensitivity, especially for samples with low viral loads. To increase the likelihood possibility of detecting vs. missing early infections in cases with high Ct values, repeated, regular testing is recommended in a mass-screening setting (67).

We also collected questionnaire responses from our participants. It is noteworthy that most of the respondents stated that PCR screening should be performed regularly to ensure a safer work environment. At the same time, the majority were willing to be tested only if screening was free or low

cost. The current questionnaire results may be informative to companies, schools, governmental organizations, hospitals, and local communities that are considering screening programs.

In conclusion, pooled RT-qPCR testing of saliva samples is effective and efficient in screening populations with relatively low prevalence. Monitoring a late amplification pattern helps increase detecting positive cases in pooled testing. Regular and frequent screening testing is generally accepted; however, financial costs could be a barrier.

Acknowledgements

The authors thank Ms. Kaori Mochida for her technical assistance; Philips Japan, Ltd. for participating in the PCR screening study, conducting the survey, and collecting and sharing the data; Editage (www.editage.com) for English language editing; Dr. Akihito Hagihara for his advice on statistical analysis and interpretation; Dr. Scott E. Woodman (Houston, TX, USA) for proofreading our manuscript, and Dr. A. Gordon Robertson (Courtenay, BC, Canada) for his advice on data visualization, data analysis, and on the manuscript. This research was conducted as part of the Keio Donner Project against COVID-19. This work was partially supported by the Keio University Global Research Institute (KGRI) Research Projects for New Coronavirus Crisis: Implementation of a Keio Model to Optimize SARS-CoV-2 PCR Tests through Systems Approach (PI: Koichi Matsuo), the Japan Agency for Medical Research and Development (AMED) (PI: Hiroshi Nishihara, Grant Number 20he1422004j0001), Grant-in-Aid for Scientific Research (C) of JSPS KAKENHI (PI: Junna Oba, Grant Number JP21K10334), and the Ministry of Education, Culture, Sports, Science and Technology of Japan (MEXT) for utilization of the university's PCR equipment. The funding agencies had no role in the study design, collection, analysis, or interpretation of data; in the writing of the report; or in the decision to submit the article for publication.

Author contributions

This study was conceptualized by Hiroshi Nishihara. Material preparation, experiments, and data collection were performed by Masae Sato, Masaki Takanashi, and Moe Yokemura. Formal analysis and investigation were performed by Junna Oba. The first draft of the manuscript was written by Junna Oba, and Hiroaki Taniguchi, Yasunori Sato and Hiroshi Nishihara commented on previous versions of the manuscript. All authors read and approved the final manuscript.

Figure Legends

Figure 1. PCR amplification curves for positive case 1 (A) and positive case 2 (B). Upper panel, N1; middle panel, N2; lower panel, internal control (IC) gene. Left column of each panel, 10-sample pools; right column of each panel, individual samples.

Figure 2. Examples of good and poor PCR amplification. (A) Good amplification with a sigmoid pattern. (B) Poor amplification lacking a sigmoid pattern.

Figure 3. Characteristics of survey participants. (A) Age group distribution with males (blue) and females (light blue). (B) Locations at the time of screening, showing prefectures with more than 10 participants.

Figure 4. Responses to the online survey.

References

1. World Health Organization. WHO Coronavirus (COVID-19) Dashboard. Available from: https://covid19.who.int/ (accessed Jan. 17, 2022).
2. Worldometer. COVID-19 coronavirus pandemic. Available from: https://www.worldometers.info/coronavirus/ (accessed Jan. 17, 2022).
3. Brogna B, Bignardi E, Brogna C, Capasso C, Gagliardi G, Martino A, et al. COVID-19 Pneumonia in Vaccinated Population: A Six Clinical and Radiological Case Series. Medicina (Kaunas). 2021;57(9).
4. Brown CM, Vostok J, Johnson H, Burns M, Gharpure R, Sami S, et al. Outbreak of SARS-CoV-2 Infections, Including COVID-19 Vaccine Breakthrough Infections, Associated with Large Public Gatherings - Barnstable County, Massachusetts, July 2021. MMWR Morb Mortal Wkly Rep. 2021;70(31):1059-62.
5. Bergwerk M, Gonen T, Lustig Y, Amit S, Lipsitch M, Cohen C, et al. Covid-19 Breakthrough Infections in Vaccinated Health Care Workers. N Engl J Med. 2021;385(16):1474-84.
6. Wu P, Liu F, Chang Z, Lin Y, Ren M, Zheng C, et al. Assessing Asymptomatic, Presymptomatic, and Symptomatic Transmission Risk of Severe Acute Respiratory Syndrome Coronavirus 2. Clin Infect Dis. 2021;73(6):e1314-e20.
7. Lee S, Meyler P, Mozel M, Tauh T, Merchant R. Asymptomatic carriage and transmission of SARS-CoV-2: What do we know? Can J Anaesth. 2020;67(10):1424-30.
8. He X, Lau EHY, Wu P, Deng X, Wang J, Hao X, et al. Temporal dynamics in viral shedding and transmissibility of COVID-19. Nat Med. 2020;26(5):672-5.
9. Nextstrain. Genomic epidemiology of novel coronavirus. Available from: https://nextstrain.org/ncov/gisaid/global (accessed Jan. 17, 2022).
10. Sethuraman N, Jeremiah SS, Ryo A. Interpreting Diagnostic Tests for SARS-CoV-2. JAMA. 2020;323(22):2249-51.
11. Koskinen A, Tolvi M, Jauhiainen M, Kekalainen E, Laulajainen-Hongisto A, Lamminmaki S. Complications of COVID-19 Nasopharyngeal Swab Test. JAMA Otolaryngol Head Neck Surg. 2021;147(7):672-4.
12. Rigante M, Picciotti PM, Parrilla C. A Nasal Complication of Nasopharyngeal Swab for Reverse Transcription Polymerase Chain Reaction (RT-PCR) Detection of SARS-CoV-2 mRNA. Cureus. 2021;13(7):e16183.
13. Foh B, Borsche M, Balck A, Taube S, Rupp J, Klein C, et al. Complications of nasal and pharyngeal swabs: a relevant challenge of the COVID-19 pandemic? Eur Respir J. 2021;57(4).
14. Azzi L, Carcano G, Gianfagna F, Grossi P, Gasperina DD, Genoni A, et al. Saliva is a reliable tool to detect SARS-CoV-2. J Infect. 2020;81(1):e45-e50.

15. Barat B, Das S, De Giorgi V, Henderson DK, Kopka S, Lau AF, et al. Pooled Saliva Specimens for SARS-CoV-2 Testing. J Clin Microbiol. 2021;59(3).
16. Williams E, Bond K, Zhang B, Putland M, Williamson DA. Saliva as a Noninvasive Specimen for Detection of SARS-CoV-2. J Clin Microbiol. 2020;58(8).
17. Nacher M, Mergeay-Fabre M, Blanchet D, Benois O, Pozl T, Mesphoule P, et al. Diagnostic accuracy and acceptability of molecular diagnosis of COVID-19 on saliva samples relative to nasopharyngeal swabs in tropical hospital and extra-hospital contexts: The COVISAL study. PLoS One. 2021;16(9):e0257169.
18. Wyllie AL, Fournier J, Casanovas-Massana A, Campbell M, Tokuyama M, Vijayakumar P, et al. Saliva or Nasopharyngeal Swab Specimens for Detection of SARS-CoV-2. N Engl J Med. 2020;383(13):1283-6.
19. Sakanashi D, Asai N, Nakamura A, Miyazaki N, Kawamoto Y, Ohno T, et al. Comparative evaluation of nasopharyngeal swab and saliva specimens for the molecular detection of SARS-CoV-2 RNA in Japanese patients with COVID-19. J Infect Chemother. 2021;27(1):126-9.
20. Herrera LA, Hidalgo-Miranda A, Reynoso-Noveron N, Meneses-Garcia AA, Mendoza-Vargas A, Reyes-Grajeda JP, et al. Saliva is a reliable and accessible source for the detection of SARS-CoV-2. Int J Infect Dis. 2021;105:83-90.
21. Hasanoglu I, Korukluoglu G, Asilturk D, Cosgun Y, Kalem AK, Altas AB, et al. Higher viral loads in asymptomatic COVID-19 patients might be the invisible part of the iceberg. Infection. 2021;49(1):117-26.
22. Sun Q, Li J, Ren H, Pastor L, Loginova Y, Madej R, et al. Saliva as a testing specimen with or without pooling for SARS-CoV-2 detection by multiplex RT-PCR test. PLoS One. 2021;16(2):e0243183.
23. Butler-Laporte G, Lawandi A, Schiller I, Yao M, Dendukuri N, McDonald EG, et al. Comparison of Saliva and Nasopharyngeal Swab Nucleic Acid Amplification Testing for Detection of SARS-CoV-2: A Systematic Review and Meta-analysis. JAMA Intern Med. 2021;181(3):353-60.
24. Nasiri K, Dimitrova A. Comparing saliva and nasopharyngeal swab specimens in the detection of COVID-19: A systematic review and meta-analysis. J Dent Sci. 2021;16(3):799-805.
25. Bastos ML, Perlman-Arrow S, Menzies D, Campbell JR. The Sensitivity and Costs of Testing for SARS-CoV-2 Infection With Saliva Versus Nasopharyngeal Swabs : A Systematic Review and Meta-analysis. Ann Intern Med. 2021;174(4):501-10.
26. Tutuncu EE, Ozgur D, Karamese M. Saliva samples for detection of SARS-CoV-2 in mildly symptomatic and asymptomatic patients. J Med Virol. 2021;93(5):2932-7.

27. Yang Q, Saldi TK, Gonzales PK, Lasda E, Decker CJ, Tat KL, et al. Just 2% of SARS-CoV-2-positive individuals carry 90% of the virus circulating in communities. Proc Natl Acad Sci U S A. 2021;118(21).
28. Nagura-Ikeda M, Imai K, Tabata S, Miyoshi K, Murahara N, Mizuno T, et al. Clinical Evaluation of Self-Collected Saliva by Quantitative Reverse Transcription-PCR (RT-qPCR), Direct RT-qPCR, Reverse Transcription-Loop-Mediated Isothermal Amplification, and a Rapid Antigen Test To Diagnose COVID-19. J Clin Microbiol. 2020;58(9).
29. To KK, Tsang OT, Yip CC, Chan KH, Wu TC, Chan JM, et al. Consistent Detection of 2019 Novel Coronavirus in Saliva. Clin Infect Dis. 2020;71(15):841-3.
30. Daniel EA, Esakialraj LB, S A, Muthuramalingam K, Karunaianantham R, Karunakaran LP, et al. Pooled Testing Strategies for SARS-CoV-2 diagnosis: A comprehensive review. Diagn Microbiol Infect Dis. 2021;101(2):115432.
31. Emmanuel JC, Bassett MT, Smith HJ, Jacobs JA. Pooling of sera for human immunodeficiency virus (HIV) testing: an economical method for use in developing countries. J Clin Pathol. 1988;41(5):582-5.
32. Lagopati N, Tsioli P, Mourkioti I, Polyzou A, Papaspyropoulos A, Zafiropoulos A, et al. Sample pooling strategies for SARS-CoV-2 detection. J Virol Methods. 2021:114044.
33. Centers for Disease Control and Prevention. COVID-19; Interim Guidance for Use of Pooling Procedures in SARS-CoV-2 Diagnostic and Screening Testing (updated June 30, 2021). Available from: https://www.cdc.gov/coronavirus/2019-ncov/lab/pooling-procedures.html (accessed Nov. 4, 2021).
34. Watkins AE, Fenichel EP, Weinberger DM, Vogels CBF, Brackney DE, Casanovas-Massana A, et al. Increased SARS-CoV-2 Testing Capacity with Pooled Saliva Samples. Emerg Infect Dis. 2021;27(4).
35. Abdalhamid B, Bilder CR, McCutchen EL, Hinrichs SH, Koepsell SA, Iwen PC. Assessment of Specimen Pooling to Conserve SARS CoV-2 Testing Resources. Am J Clin Pathol. 2020;153(6):715-8.
36. Bish DR, Bish EK, El-Hajj H, Aprahamian H. A robust pooled testing approach to expand COVID-19 screening capacity. PLoS One. 2021;16(2):e0246285.
37. Ben-Amotz D. Optimally pooled viral testing. Epidemics. 2020;33:100413.
38. Aragon-Caqueo D, Fernandez-Salinas J, Laroze D. Optimization of group size in pool testing strategy for SARS-CoV-2: A simple mathematical model. J Med Virol. 2020;92(10):1988-94.
39. Oba J, Taniguchi H, Sato M, Takamatsu R, Morikawa S, Nakagawa T, et al. RT-PCR Screening Tests for SARS-CoV-2 with Saliva Samples in Asymptomatic People: Strategy to Maintain Social and Economic Activities while Reducing the Risk of Spreading the Virus. Keio J Med. 2021;70(2):35-43.

40. Mendoza RP, Bi C, Cheng HT, Gabutan E, Pagaspas GJ, Khan N, et al. Implementation of a pooled surveillance testing program for asymptomatic SARS-CoV-2 infections in K-12 schools and universities. EClinicalMedicine. 2021;38:101028.
41. Wilmes P, Zimmer J, Schulz J, Glod F, Veiber L, Mombaerts L, et al. SARS-CoV-2 transmission risk from asymptomatic carriers: Results from a mass screening programme in Luxembourg. Lancet Reg Health Eur. 2021;4:100056.
42. Denny TN, Andrews L, Bonsignori M, Cavanaugh K, Datto MB, Deckard A, et al. Implementation of a Pooled Surveillance Testing Program for Asymptomatic SARS-CoV-2 Infections on a College Campus - Duke University, Durham, North Carolina, August 2-October 11, 2020. MMWR Morb Mortal Wkly Rep. 2020;69(46):1743-7.
43. Pharmaceuticals and Medical Devices Agency. PMDA's Efforts to Combat COVID-19. Available from: https://www.pmda.go.jp/english/about-pmda/0002.html (accessed Dec. 12, 2021).
44. Centers for Disease Control and Prevention. Research use only 2019-novel coronavirus (2019-nCcoVv) real-time RT-PCR primers and probes. Available from: https://www.cdc.gov/coronavirus/2019-ncov/lab/rt-pcr-panel-primer-probes.html (accessed Aug. 10, 2021).
45. Zhen W, Berry GJ. Development of a New Multiplex Real-Time RT-PCR Assay for Severe Acute Respiratory Syndrome Coronavirus 2 (SARS-CoV-2) Detection. J Mol Diagn. 2020;22(12):1367-72.
46. Lopez Bernal J, Andrews N, Gower C, Gallagher E, Simmons R, Thelwall S, et al. Effectiveness of Covid-19 Vaccines against the B.1.617.2 (Delta) Variant. N Engl J Med. 2021;385(7):585-94.
47. Regev-Yochay G, Amit S, Bergwerk M, Lipsitch M, Leshem E, Kahn R, et al. Decreased infectivity following BNT162b2 vaccination: A prospective cohort study in Israel. Lancet Reg Health Eur. 2021;7:100150.
48. Singanayagam A, Hakki S, Dunning J, Madon KJ, Crone MA, Koycheva A, et al. Community transmission and viral load kinetics of the SARS-CoV-2 delta (B.1.617.2) variant in vaccinated and unvaccinated individuals in the UK: a prospective, longitudinal, cohort study. Lancet Infect Dis. 2021.
49. Seppala E, Veneti L, Starrfelt J, Danielsen AS, Bragstad K, Hungnes O, et al. Vaccine effectiveness against infection with the Delta (B.1.617.2) variant, Norway, April to August 2021. Euro Surveill. 2021;26(35).
50. Andrews N, Tessier E, Stowe J, Gower C, Kirsebom F, Simmons R, et al. Vaccine effectiveness and duration of protection of Comirnaty, Vaxzevria and Spikevax against mild and severe COVID-19 in the UK. medRxiv. 2021:2021.09.15.21263583.

51. Bar-On YM, Goldberg Y, Mandel M, Bodenheimer O, Freedman L, Kalkstein N, et al. Protection of BNT162b2 Vaccine Booster against Covid-19 in Israel. N Engl J Med. 2021;385(15):1393-400.
52. Taylor L. Why scientists worldwide are watching UK COVID infections. Nature. 2021;599(7884):189-90.
53. Taylor L. Covid-19: Omicron drives weekly record high in global infections. BMJ. 2022;376:o66.
54. Araf Y, Akter F, Tang YD, Fatemi R, Parvez SA, Zheng C, et al. Omicron variant of SARS-CoV-2: Genomics, transmissibility, and responses to current COVID-19 vaccines. J Med Virol. 2022.
55. Karim SSA, Karim QA. Omicron SARS-CoV-2 variant: a new chapter in the COVID-19 pandemic. Lancet. 2021;398(10317):2126-8.
56. Siegel JD, Rhinehart E, Jackson M, Chiarello L, Health Care Infection Control Practices Advisory C. 2007 Guideline for Isolation Precautions: Preventing Transmission of Infectious Agents in Health Care Settings. Am J Infect Control. 2007;35(10 Suppl 2):S65-164.
57. Yelin I, Aharony N, Tamar ES, Argoetti A, Messer E, Berenbaum D, et al. Evaluation of COVID-19 RT-qPCR Test in Multi sample Pools. Clin Infect Dis. 2020;71(16):2073-8.
58. Chong BSW, Tran T, Druce J, Ballard SA, Simpson JA, Catton M. Sample pooling is a viable strategy for SARS-CoV-2 detection in low-prevalence settings. Pathology. 2020;52(7):796-800.
59. Chen F, Geng Z, Wang J, Liuchang W, Huang D, Xu Y, et al. Comparing two sample pooling strategies for SARS-CoV-2 RNA detection for efficient screening of COVID-19. J Med Virol. 2021;93(5):2805-9.
60. Thanh TT, Nhan NTT, Mai HK, Trieu NB, Huy LX, Thuy HTT, et al. The Application of Sample Pooling for Mass Screening of SARS-CoV-2 in an Outbreak of COVID-19 in Vietnam. Am J Trop Med Hyg. 2021.
61. More S, Narayanan S, Patil G, Ghosh P, Pushparaj S, Cooper E, et al. Pooling of Nasopharyngeal Swab Samples To Overcome a Global Shortage of Real-Time Reverse Transcription-PCR COVID-19 Test Kits. J Clin Microbiol. 2021;59(4).
62. Praharaj I, Jain A, Singh M, Balakrishnan A, Dhodapkar R, Borkakoty B, et al. Pooled testing for COVID-19 diagnosis by real-time RT-PCR: A multi-site comparative evaluation of 5- & 10-sample pooling. Indian J Med Res. 2020;152(1 & 2):88-94.
63. Griesemer SB, Van Slyke G, St George K. Assessment of Sample Pooling for Clinical SARS-CoV-2 Testing. J Clin Microbiol. 2021;59(4).
64. Mohanty S, Ravindra A, Gupta K, Hallur V, Behera B, Mahaptra A, et al. Intricacies in characterizing positivity in pooled sample testing for SARS-CoV-2. J Med Virol. 2021;93(5):2799-804.

65. Ochert AS, Boulter AW, Birnbaum W, Johnson NW, Teo CG. Inhibitory effect of salivary fluids on PCR: potency and removal. PCR Methods Appl. 1994;3(6):365-8.
66. Lalli MA, Langmade JS, Chen X, Fronick CC, Sawyer CS, Burcea LC, et al. Rapid and Extraction-Free Detection of SARS-CoV-2 from Saliva by Colorimetric Reverse-Transcription Loop-Mediated Isothermal Amplification. Clin Chem. 2021;67(2):415-24.
67. Mina MJ, Parker R, Larremore DB. Rethinking Covid-19 Test Sensitivity - A Strategy for Containment. N Engl J Med. 2020;383(22):e120.
68. Larremore DB, Wilder B, Lester E, Shehata S, Burke JM, Hay JA, et al. Test sensitivity is secondary to frequency and turnaround time for COVID-19 screening. Sci Adv. 2021;7(1).

Click here to access/download;Figure;Figure 1.pdf

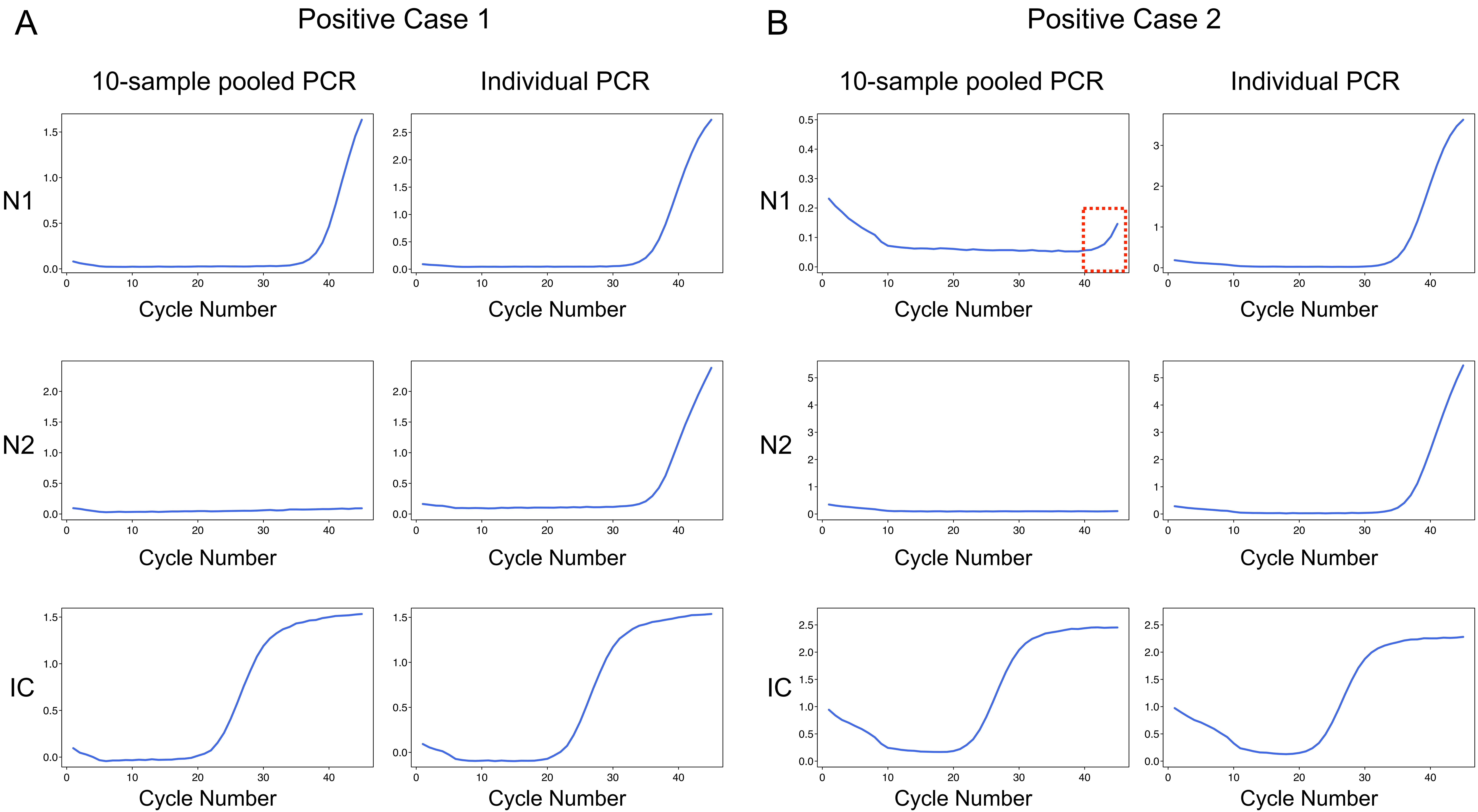

Figure 2

Click here to access/download;Figure;Figure_2_Good and Poor PCR curves.pdf

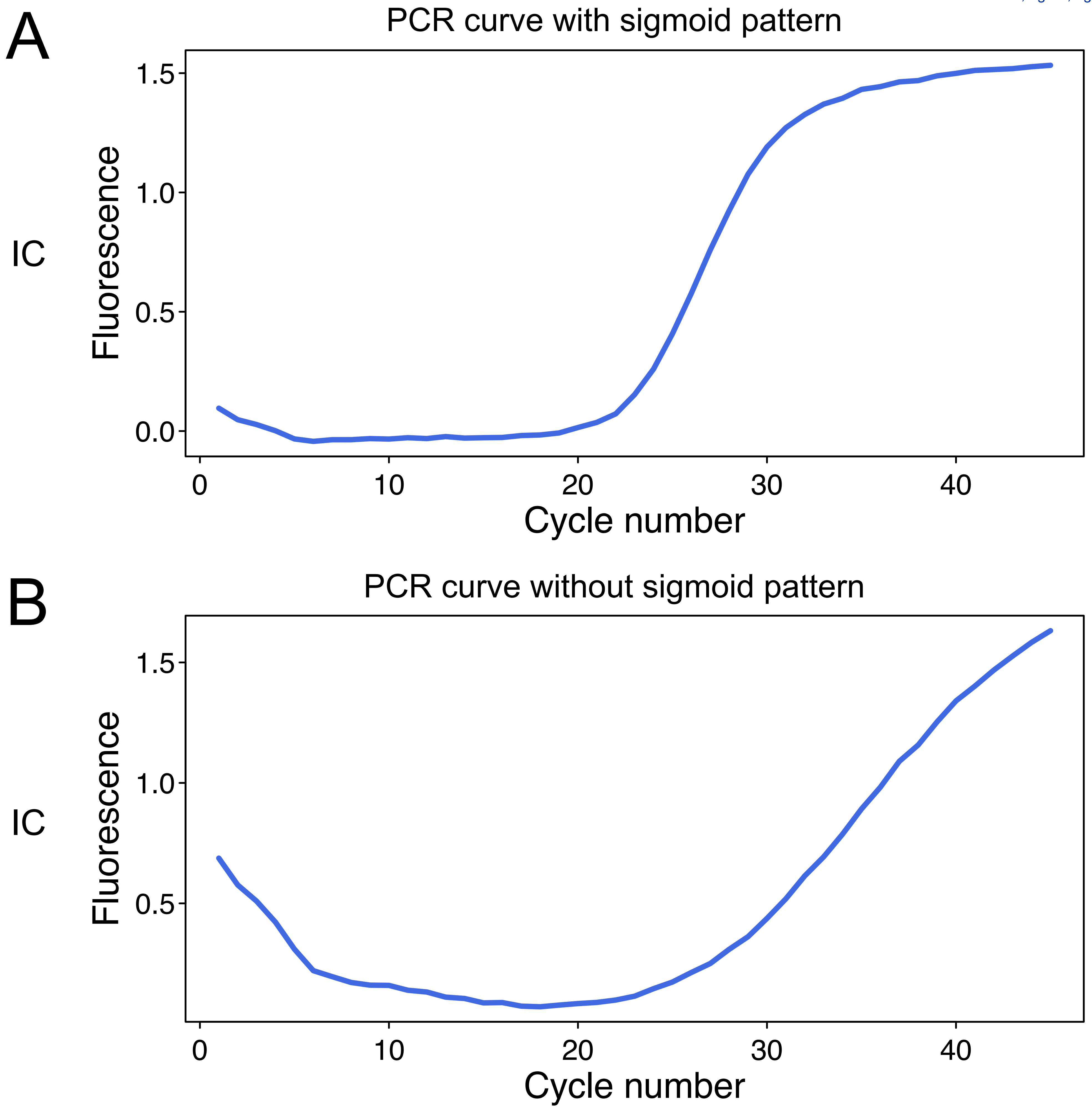

Click here to access/download;Figure;Figure 3_Age and Japan Map.pdf

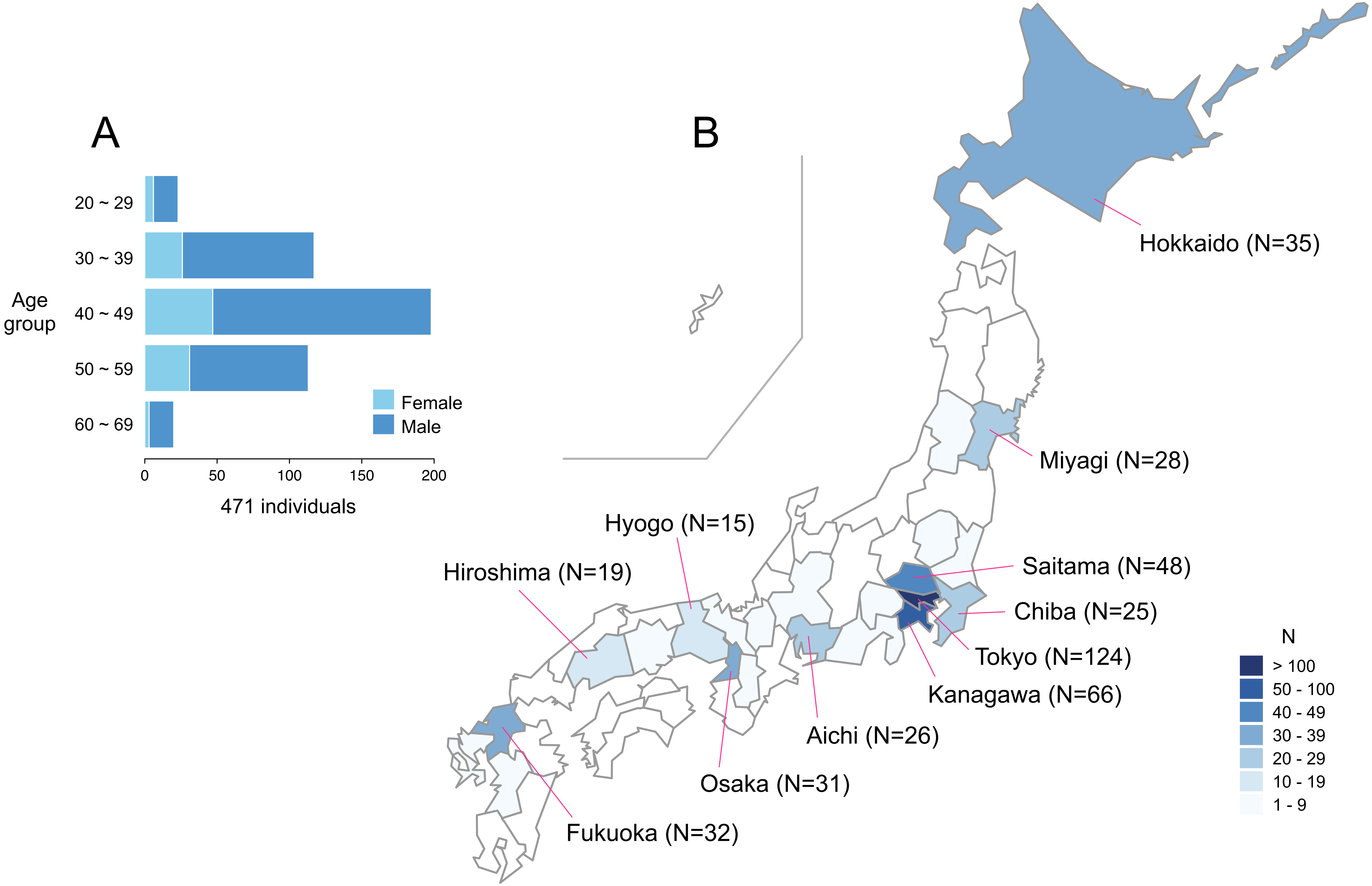

Figure 4

Click here to access/download;Figure;Figure 4_Questionnaire result plots.pdf

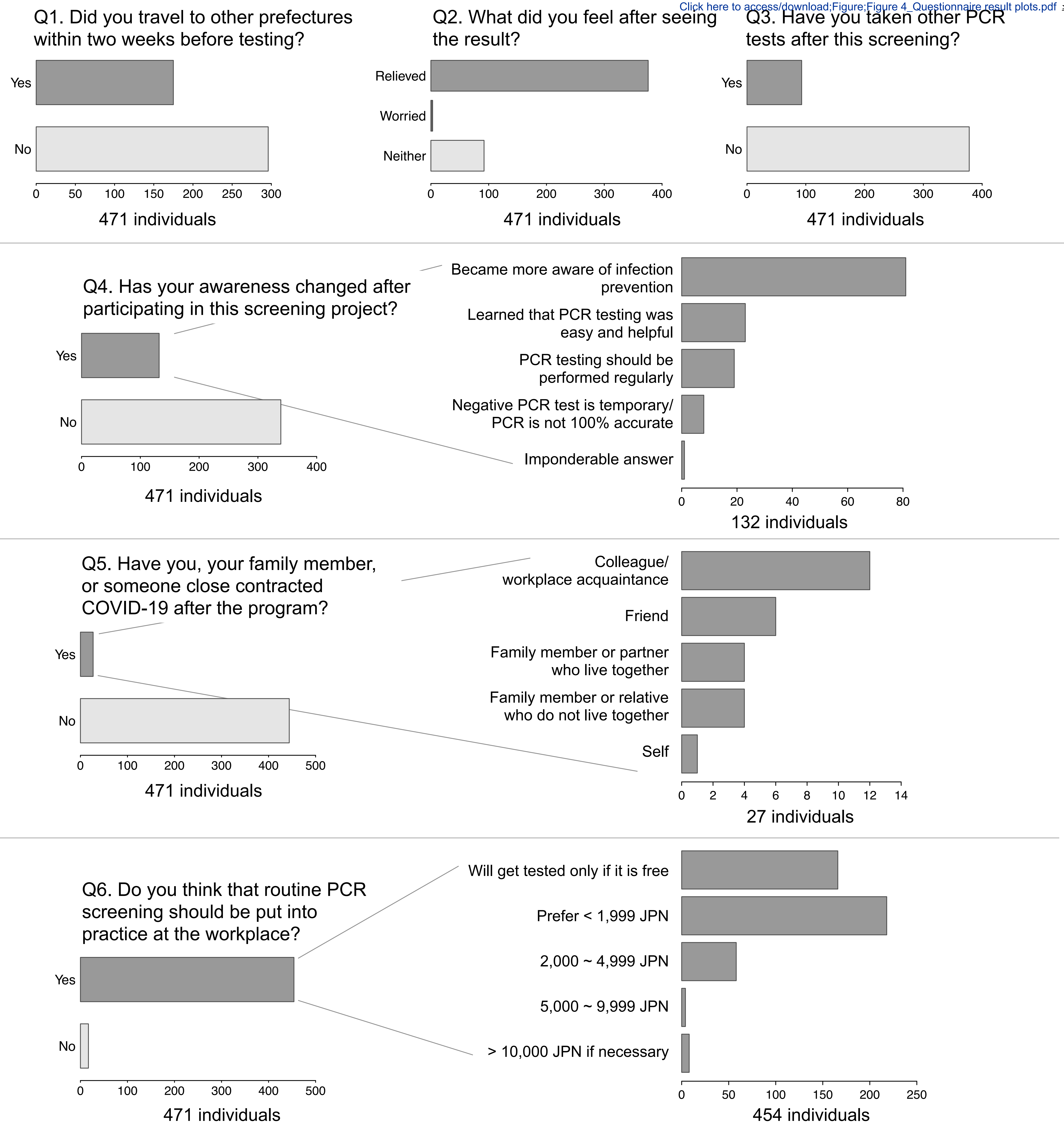

Click here to access/download
Supporting Information
Supplementary_tables_2022.1.24.xlsx
